# Automated machine learning to foster AI development processes: an explorative study

**DOI:** 10.3389/frai.2026.1867883

**Published:** 2026-10-05

**Authors:** Anna Katharina Polzer, Stefan Thalmann

**Affiliations:** Department of Operations and Information Systems, University of Graz, Graz, Austria

**Keywords:** AI development processes, AutoML, boundary object, human-centered AutoML, interdisciplinary collaboration, low-code/no-code AI

## Abstract

**Introduction:**

Communication barriers between citizen developers and AI experts are a serious challenge for AI system development in the age of No and Low Code (LCNC) AI development. Automated Machine Learning (AutoML) has emerged as a promising approach to democratize AI development by enabling non-technical stakeholders to participate in the creation of AI systems. However, its role in supporting collaboration between citizen developers and AI experts remains underexplored.

**Methods:**

This study examines AI experts' perceptions of AutoML's potential as a boundary object that facilitates communication and collaboration between citizen developers and AI experts. An exploratory qualitative interview study was conducted with 14 AI experts from various industries to examine their perceptions of how GUI-based low- or no-code AutoML prototypes may support collaboration.

**Results:**

The findings indicate that AutoML can provide a shared frame of reference that supports knowledge exchange, mutual understanding, and interdisciplinary teamwork. Interviewees highlighted that AutoML-generated insights and early-stage prototypes facilitate iterative, feedback-driven development processes and help overcome data and modeling barriers. However, the effective integration of AutoML requires appropriate governance structures, including clear responsibilities, guided processes, and structured stakeholder interaction.

**Discussion:**

This exploratory study highlights AI experts' perceptions that AutoML may serve not only as a technological tool but also as a potential facilitator of collaboration in AI development. However, democratizing AI development requires more than accessible tools alone and depends on well-managed communication and engagement processes. The study contributes to a deeper understanding of AutoML's role as a boundary object and highlights the need for further research into its responsible and inclusive deployment.

## 1 Introduction

AI adoption often falls short of expectations, as many organizations still struggle to move beyond pilot projects and successfully scale AI into real business operations (Haefner et al., 2023). One critical barrier for broader AI adoption is the lack of AI skills within organizations (Enholm et al., 2021). This knowledge gap is particularly challenging in the development of organization-specific AI systems, where domain expertise is essential for identifying relevant problems, understanding organizational requirements, and assessing whether a system is meaningful in its application context. Developing such systems requires collaboration between domain experts and AI experts. However, this collaboration can be challenging because the two groups contribute different forms of expertise and may not share the same language, assumptions, or priorities. Domain experts understand the specific business needs but lack AI expertise, while AI experts have the technical skills but are often unfamiliar with the domain in which the AI system will be applied (Zöller and Huber, 2021).

Development practices for AI systems remain an evolving field shaped by increasing technical and organizational complexity. Many challenges are domain-specific and data-related, highlighting the need for approaches that support communication and collaboration between domain and AI experts (Martínez-Fernández et al., 2022). Low-code or no-code (LCNC) tools for AI development have emerged as promising approaches to overcoming barriers to collaboration between AI and domain experts (Cabot and Clariso, 2023). By simplifying the development of AI solutions, these tools enable domain experts to engage more actively in their creation and support the democratization of AI. In particular, Automated Machine Learning (AutoML) automates key stages of machine learning development, accelerating AI adoption and enabling new application areas (Hutter et al., 2019). AutoML also provides early insights into machine learning capabilities, supporting more informed experimentation and decision-making (Wang et al., 2019). This development has led to the emergence of so-called citizen developers, a term commonly used to describe non-technical employees who are empowered by LCNC platforms to build applications or AI models without relying on AI experts (Lotfian et al., 2021; Binzer et al., 2024).

This study thus focuses on citizen developers' perceptions of how LCNC AutoML tools may influence coordination with AI experts in AI system development. Rather than addressing the development of new AI architectures or foundation models, the study investigates how AutoML prototypes could support communication, coordination, and shared understanding in developing context- and organization-specific AI systems, in which technical solutions must align with local data, work practices, requirements, and business goals.

The democratization of AI, driven by LCNC AutoML, raises broader questions regarding collaboration, decision-making, and accountability in AI system development. While much of the existing research has focused on how AutoML can enhance the technical modeling phase (Barbudo et al., 2023), less is known about how it shapes interaction between citizen developers and AI experts. Existing work has focused mainly on technology and efficiency and researchers are calling for more research on the effects on stakeholders (Karmaker et al., 2021; Al Alamin and Uddin, 2024). In this regard, boundary object theory is commonly used to analyze development processes from a multi-stakeholder perspective (Holtmann et al., 2025). Boundary objects are artifacts or concepts that facilitate communication and collaboration between different groups, providing a shared frame of reference even when those groups interpret them differently (Star and Griesemer, 1989). This paper aims to explore AI experts' perceptions of how AI prototypes developed with AutoML may function as a boundary object in AI development processes, and the anticipated ways it could support coordination between citizen developers and AI experts. The research question guiding this study is:

*How do AI experts perceive the potential role of low-code/no-code (LCNC) AutoML tools in supporting collaboration with citizen developers during AI development processes?*

To address the research question, we conducted exploratory interviews with 14 AI experts and practitioners. Participants were professionals involved in the development and/or deployment of AI systems who also contributed to requirements alignment and communication with domain stakeholders.

## 2 Background

### 2.1 AutoML to democratize AI development

Limited knowledge among employees and users affects multiple aspects of AI development, including understanding AI capabilities and managing expectations (Baier et al., 2019), trust and acceptance (Enholm et al., 2021), and, in particular, collaboration and communication (Westenberger et al., 2022). Recent research further highlights that resistance to AI adoption, gaps in digital literacy, and insufficient organizational readiness can significantly hinder effective knowledge management and AI integration (Gelashvili-Luik et al., 2025). Additionally, the absence of domain knowledge among AI experts can pose a barrier (Papagiannidis et al., 2023). Sjödin et al. (2021) suggest that organizations should not only focus on developing AI capabilities but also on democratizing them across the entire organization, enabling employees to act as citizen developers and explore and implement promising use cases themselves (Johnson et al., 2022).

AutoML tools are central to these democratization efforts, simplifying the AI development process and making it more accessible (Sundberg and Holmström, 2023). AutoML, a subfield of AI research, aims to automate various stages of AI system design and development (Al Alamin and Uddin, 2024; Hutter et al., 2019). In practice, you can find AutoML libraries such as AutoKeras for data scientists and AI experts who require substantial coding knowledge, as well as LCNC AutoML tools designed for citizen developers, such as KNIME or Google Vertex, that rely on graphical user interfaces. By reducing the need for programming, AutoML empowers citizen developers to independently create AI applications, even without data science or AI knowledge (He et al., 2021). This focus on lowering technical barriers is reflected in the growing popularity of LCNC AutoML tools, especially from commercial providers (Al Alamin and Uddin, 2024).

However, traditional AI development still faces challenges and insights from traditional software engineering processes cannot be transferred without adjustments to the development of AI systems (Martínez-Fernández et al., 2022). Unique characteristics of AI systems need to be considered, which range from the probabilistic nature of learning-based AI systems (Dolata and Crowston, 2024), the necessity for high-quality data for AI system development (Wan et al., 2020) to the challenges in testing and validation of such systems (Baier et al., 2019; Wan et al., 2020). These characteristics pose challenges throughout the development process and make domain expertise essential for building AI solutions that are both technically sound and meaningful within their organizational context.

Several well-established challenges underscore the importance of domain knowledge throughout AI system development. At the start of AI system development, insufficient domain knowledge can hinder the identification of feasible use cases aligned with organizational requirements, leading to poorly specified problems, unrealistic expectations, and overlooked regulatory or organizational constraints (Bauer et al., 2020; Westenberger et al., 2022; Papagiannidis et al., 2023). During data handling, an essential part of AI system development, challenges lie in the assessment of meaningful, representative, and suitable data for the intended context (Westenberger et al., 2022). Data work is often iterative, involving the integration and cleaning of sources, relevance decisions, and annotation (Ziv and Nakash, 2025). Without domain-specific input and coordination across teams, data quality problems undermine model reliability, fairness, traceability, and compliance (Ziv and Nakash, 2025; Zöller and Huber, 2021). Additionally, domain knowledge is required to interpret performance metrics, error patterns, and model behavior. Without clear communication between technical and domain stakeholders, even technically high-performing models may be considered unusable or of limited practical value (Westenberger et al., 2022; Papagiannidis et al., 2023). When developing context-specific AI systems, more cyclical development processes are often preferred, emphasizing frequent coordination between domain and AI experts (Laato et al., 2023; Nascimento et al., 2019). Although more detailed AI development frameworks distinguish a larger number of phases and subtasks (Hill et al., 2016; Amershi et al., 2019; Studer et al., 2021), the four-stage process proposed by Nascimento et al. (2019) - *problem understanding, data handling, model building, and model monitoring*- provides a suitable level of abstraction for this study. As illustrated in Figure 1, these iterative stages rely heavily on communication between domain and AI experts. Data-centric perspectives further emphasize that the relevance and usefulness of data depend on its origin and intended use; consequently, data must be revisited throughout the AI lifecycle as domain conditions and organizational requirements change, to determine whether a technically plausible prototype addresses the right organizational problems (Ziv and Nakash, 2025). Domain knowledge can therefore enhance AI experts' productivity (Chapetta and Travassos, 2020), although collaboration remains challenging when knowledge gaps undermine trust and a shared understanding of AI capabilities and outcomes (Piorkowski et al., 2021).

**Figure 1:**
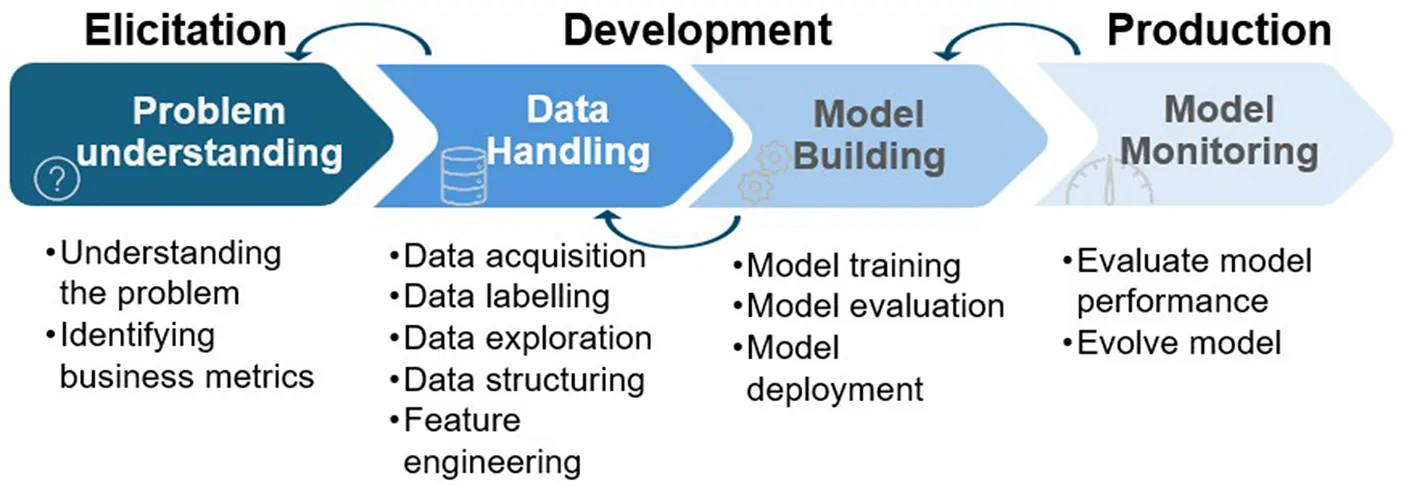
AI development process according to Nascimento et al. (2019).

Expert-driven AI development typically involves several interdependent roles, including data engineers, data scientists, ML engineers, DevOps engineers, and MLOps engineers (Ziv and Nakash, 2025). In contrast, Karmaker et al. (2021) distinguish two primary AutoML user groups: citizen developers with deep domain knowledge but limited ML expertise, and AI experts proficient in AI development but less familiar with the domain. Depending on users' skill levels, AutoML tools and frameworks differ in their degree of automation, ranging from programming libraries that automate parts of the ML pipeline to user-friendly, drag-and-drop applications that support the full AI development lifecycle (Calefato et al., 2023). These tools assist with tasks such as data preparation, feature engineering, hyperparameter optimization, model selection, and end-to-end AI system development (He et al., 2021).

Despite the maturity of AutoML tools such as Google AutoML, KNIME, and H2O, research on their use remains limited. In particular, LCNC AutoML tools enable citizen developers to formulate potential use cases, explore data through guided workflows, and develop initial prototypes (Wang et al., 2019). At the same time, AutoML does not replace human expertise: skilled users remain essential for contributing domain knowledge, identifying tool limitations, safeguarding outputs, and adapting inputs to the requirements of the application context (Xin et al., 2021; Quaranta et al., 2025). Karmaker et al. (2021) argue that integrating AutoML into AI development can substantially enhance efficiency and address challenges in expert-driven AI development by improving communication and collaboration. However, citizen developers face serious challenges in assessing the quality of their AI models and in coordinating with IT experts to ensure successful integration into business processes, which is considered a major challenge (Karmaker et al., 2021). Despite its growing importance, little is known about how AutoML can enhance communication and collaboration between citizen developers and AI experts. AutoML tools could serve as boundary objects that facilitate alignment and knowledge exchange, especially in the early, communication-intensive development phases, yet this role remains underexplored (Truss and Schmitt, 2024). Addressing this issue calls for more in-depth research.

### 2.2 AutoML as boundary objects in AI development

Boundary objects, first described by Star and Griesemer (1989), are artifacts or constructs that maintain a shared understanding across different groups while remaining flexible enough to adapt to local contexts and to facilitate alignment and knowledge exchange. To facilitate knowledge exchange across boundaries in software development, boundary objects can take many forms, such as prototypes, data schemata, development sketches, or modeling tools (Bergman et al., 2007; Holtmann et al., 2025). Such boundary objects serve as moderation, mediation, evaluation and alignment artifacts between stakeholder groups (Jalowski et al., 2022).

Wenger, (2000) extended this idea, describing them not just as tangible artifacts, but also as elements that enable learning and communication across communities of practice. He identifies several potential forms of boundary objects, including artifacts, discourse, and processes. Artifacts, such as prototypes or diagrams, provide a concrete shared reference for participants from different communities. Discourse supports the development of a common language through which these participants can communicate and negotiate shared meanings. Additionally, Wenger recognizes shared processes, such as explicit routines and procedures, as another form of boundary object that can support coordination across boundaries (Wenger, 2000). However, boundary objects do not necessarily create consensus. They can make differing interpretations and disagreements visible for discussion. In LCNC AutoML, shared workflows and model outputs may support dialogue, but also risk oversimplification, making expert facilitation and review essential.

Knowledge boundaries in IT system development processes have thus been studied in various contexts (Kotlarsky et al., 2015; Foerderer et al., 2019) and it has been shown that positive effects from knowledge integration can be achieved through the utilization of boundary objects (Patnayakuni et al., 2007). Boundary objects have been recognized as valuable in design and development processes for facilitating knowledge transfer, improving organizational learning, supporting collaboration, enhancing efficiency and decision-making, adapting to diverse needs, promoting knowledge retention, and addressing communication gaps (Butters and Duryan, 2019). In software development, artifacts such as specifications and project management tools help address technical and cultural differences across teams, though they can also spark conflict (Barrett and Oborn, 2010). In the context of AI and algorithmic fairness, boundary objects have also been proposed as mechanisms to facilitate interdisciplinary collaboration and shared understanding across domains with differing perspectives and experiences (Kontiainen et al., 2022). While these objects can enhance collaboration, they can also contribute to conflict. Effective use, therefore, depends on tacit knowledge and the interactive use of cultural and functional cues to bridge knowledge boundaries and build shared understanding (Barrett and Oborn, 2010).

Further, McLoughlin et al. (2016) examined boundary objects in health information infrastructure projects, showing that they foster collaboration and co-design among diverse stakeholders but can fail when applied through rigid, top-down approaches that misalign with existing practices. Similarly, it has been demonstrated that software prototypes and other artifacts, serving as boundary objects, mediate knowledge exchange and coordination across different stages of software development (Huber et al., 2020; Zaitsev et al., 2020).

While the role of boundary objects in software development has been explored, their application in AI development remains underexplored. AI development shares some challenges with conventional software development, such as knowledge boundaries, but also faces unique AI-specific hurdles that demand tailored approaches (Misra et al., 2023). Boundary objects known from software development often fail to address these challenges, highlighting the need for AI-specific boundary objects. Notably, the lack of suitable boundary objects for collaboration in AI prototyping has been identified as a major challenge (Truss and Schmitt, 2024). AutoML shows promise as a potential boundary object within AI development, offering opportunities to bridge knowledge gaps and enhance collaboration. However, its role in this context remains relatively unexplored, underscoring the need for further research.

## 3 Study design

This study explores AI experts' perceptions of the potential role of AutoML in AI development, focusing on collaboration and communication with citizen developers. Due to the limited related work and, consequently, the absence of testable hypotheses, we adopted a qualitative, exploratory research approach using semi-structured interviews (Mingers, 2001).

Due to the exploratory nature of the study, we targeted interview participants who are actively involved in the AI development process and are in roles that involve direct communication with citizen developers. Participants were recruited using a snowball approach, starting with potential interview partners in our own networks and then expanding to the interviewees' professional networks. The goal was to get a broad overview without focusing on a specific industry sector or type of organization. Accordingly, we interviewed 14 participants from various industries. An overview of the interviewees is provided in Table 1. Twelve out of 14 interviewees were male (85.71%) and two were female (14.29%). Interviews were conducted in English (*n* = 6) or German (*n* = 8), either online or in person, and lasted an average of 45 min.

**Table 1:** Overview of interview participants.

ID	Participant role	Years (in data science/ML)	Organization domain	Duration (minutes)
P1	CEO and founder	10+	AI software development	48
P2	Head of data science	5	Consulting	53
P3	ML consultant	2	Consulting	34
P4	Senior data scientist	5	Consulting	35
P5	Head of data science & big data	9	Consulting	39
P6	Head of data science	12	Consulting	44
P7	Data engineer/AI expert	8	Energy provider	58
P8	Data scientist & BI engineer	3	Insurance	37
P9	Solution architect	5	Manufacturing industry	29
P10	Data architect	7	Manufacturing industry	38
P11	Lead researcher	5	Research	82
P12	Head of data science	9	Research	47
P13	Data scientist & researcher	4	Research	58
P14	Data scientist & researcher	5	Research	44

Participants are referred to as AI experts because they were selected not only for their technical involvement in AI development, but also for their regular interaction with clients, customers, and other business stakeholders. Although their roles ranged from data engineering and data science to consulting, research, architecture, and leadership, all participants performed boundary-spanning work: translating requirements, discussing data and use cases, and coordinating between technical teams and domain experts. The study therefore focuses on their experience of this communication- and coordination-intensive work across the AI development process.

We created an interview guideline based on a literature review, which included themes surrounding AI development projects and AutoML use and integration. A pilot interview was conducted to refine the clarity and relevance of the questions; its data were excluded from the analysis. In total, we conducted 14 interviews (two in person, twelve online). All participants provided written consent before participating.

The interviews began with general questions about the participants and their respective organizations, including their roles and responsibilities. Following this, participants were asked to reflect on the challenges they encountered during collaboration with AI citizen developers, the mitigation strategies they had adopted within their organizations, the effectiveness of these strategies, and the current state of AI development practices. Participants then reflected on their awareness, understanding, and use of AutoML in general, and of its use by citizen developers. The discussions enabled participants to elaborate freely on their experiences, perspectives on AI development challenges, and views on the role of AutoML tools, such as Google AutoML and Microsoft Azure AutoML, in collaboration with citizen developers. Participants were provided with a definition of LCNC AutoML to establish a shared understanding of the study's key concepts. They were then invited to reflect on potential use cases for integrating these tools across the AI development process and to assess their anticipated impact. The semi-structured format allowed for consistent discussions of key themes.

All interviews were transcribed using Trint, manually verified for accuracy, and pseudonymized before analysis in MAXQDA. We applied informed content analysis, as it allows using initial categories as structuring elements derived from existing literature and serving as a foundation for the study, while remaining open to emerging insights (Patton, 2015). Guided by prior research on AI development challenges, initial codes were developed and iteratively refined following Mayring's approach (Mayring, 2022). After reviewing the first five interviews, codes were adjusted to better capture nuances and grouped into broader categories through constant comparison.

During coding, three overarching themes emerged, each connected to different aspects of AI development: (1) organizational and infrastructure challenges, which encompassed management and lifecycle issues, data handling structures, and regulatory concerns (2) process-related factors especially in regard to human involvement, such as expectations around AI capabilities, knowledge transfer, communication difficulties, and strategies to overcome them and (3) perceptions of AutoML, including user requirements, integration into existing development workflows, and both its opportunities and associated risks.

While the initial coding phase provided insight into challenges in AI development and AutoML integration, the analysis did not yield a comprehensive answer to our research questions. Thus, we decided to refine the coding scheme in a second step to mitigate challenges in AI development projects. Based on the insights gained, this phase emphasized two main perspectives: AutoML's technical capabilities and its role as a boundary object.

To provide a coherent structure, the codes were aligned with the phases of AI development described by Nascimento et al. (2019). Based on this coding scheme, the identified themes were then compared with the theoretical framework of Boundary Object theory (Miles and Huberman, 2009). This analysis explored how AutoML may serve as a boundary object in AI development, through its role as a shared artifact, discourse, and process (Wenger, 2000).

The primary concepts, overarching themes, and selected quotes were transcribed into English for clarity and communication. This systematic and iterative process ensured the robustness and credibility of the findings while also enabling researchers to explore how participants described AutoML's potential role across phases of AI development, drawing on existing knowledge from Boundary Object Theory.

## 4 Findings

We used the AI development process according to Nascimento et al. (2019) to structure the findings. Our study focuses on citizen developers' use of AutoML to create early prototypes to explore and validate use cases, rather than to develop AI solutions for ongoing operational use. We therefore exclude model monitoring, as it was neither a central focus of the interview questions nor examined in sufficient empirical depth. Accordingly, the analysis does not address the creation of novel AI architectures or LLMs, nor the technical details of production implementation. Instead, it examines the AutoML prototype as a boundary object at the interface between citizen developers and AI experts.

We differentiate between AutoML, a tool with technical capabilities for addressing challenges, and the AutoML prototype, a boundary object that participants perceived as potentially supporting collaboration, communication, and learning between citizen developers and AI experts. As such, the AutoML prototype combines the AI use case idea, underlying data, and the technical solution generated by the AutoML tool. For each phase, we explore how incorporating such a prototype could help address coordination challenges, while also considering how poor implementation could reinforce existing boundaries or create new ones.

### 4.1 Problem understanding

AutoML prototypes can considerably impact the problem understanding phase by serving as boundary objects that facilitate communication and collaboration. The standardized workflows and representations by AutoML tools were perceived to promote a common language, minimizing misunderstandings and enhancing effective communication. Participants highlighted that the problem understanding phase often involves extensive back-and-forth communication between domain and AI experts. Drawing on experience communicating with industry partners, P13 described AutoML as a possible way to make early AI ideas more accessible to business stakeholders:

“*And I think it could be also a good way to communicate with the domain experts. [...] for the customers with a bit of knowledge about machine learning, it's going to be a good tool for them to make some proof of concepts.”* (P13)

This account suggests that AutoML prototypes may help stakeholders explore and discuss potential use cases through an early proof of concept. They help citizen developers gain clarity on how their concepts could materialize in an AI system, while AI experts gain insights into applying standard ML approaches to specific domains. The prototype was therefore considered a starting point for discourse on more complex approaches.

As a shared artifact, the AutoML prototype was perceived as also structuring the surrounding discourse and fostering mutual learning. Participants all highlighted that problem formulation is a time-intensive process, mainly due to communication challenges and a lack of mutual understanding: citizen developers often struggle to articulate their expectations for AI solutions because of their limited understanding of what AI can realistically achieve. At the same time, AI experts may lack the domain knowledge needed to identify viable use cases and associated dependencies, such as data requirements. As one AI expert explained:

“*And here you have to talk with people, you need to be communicative, you need to sit with people and just chat about what they are doing, what even exists. It is really like searching in the dark...”* (P2)

By providing citizen developers with a concrete interface for engaging with a model, the participants suggested that AutoML may help bridge these knowledge gaps and support the alignment of requirements. Similarly, P10, an AI practitioner in the IT department of an international manufacturing company who regularly worked with internal business clients on AI systems, emphasized the value of enabling citizen developers to work directly with a model:

“*To involve them [citizen developers], there is nothing better than providing them an interface to train their own model.”* (P10)

AutoML may consequently support problem formulation and project scoping. Accessible, hands-on platforms, pre-defined workflows and intuitive interfaces can lower entry barriers and enable citizen developers to experiment with AI system development using their own data, fostering familiarity with the technology. This helps citizen developers to better understand AI's potential and communicate contextual insights more effectively. One interviewee noted the enthusiasm among citizen developers for such tools, remarking:

“*I'm very sure the people using it would love something like that. Very, very sure, because many times I even work with them together on smaller data sets where we don't use Azure machine learning, but we use local instances of maybe some Python data science toolkit.”* (P7)

Participants therefore regarded AutoML as a means to improve coordination in early-stage project discussions. As one interviewee affirmed:

“*I am 100% convinced that AutoML can help in the definition of scope”* (P12)

In this sense, AutoML prototypes provide a tangible representation of a potential AI use case. Although AutoML tools do not encompass all AI capabilities, they can provide a valuable starting point by linking domain knowledge to a prototype comprehensible to both domain and AI experts. Standardization may further reduce the effort required from AI specialists for routine tasks, allowing them to focus on fine-tuning and more complex challenges.

However, these benefits depend on AutoML being integrated as a boundary object rather than used as a stand-alone solution. If the AutoML prototypes fail to support collaboration and shared understanding, they could reinforce knowledge gaps and hinder productive discourse. Interviewees voiced concerns that unsupervised use by citizen developers may lead to fixed ideas about what is possible and how AI projects should be approached, adopting a rigid perspective. This may lead to “blind trust” in the prototype and limit openness to alternative solutions. As one interviewee explained:

“*But yes, that is again a double-edged sword. When you pin yourself down to one idea and it needs to stay within those exact limits, of course the situation can arise in which you say Yes, but it does not work within this scope, in fact you can prove that it does not work”* (P12)

What the interviewee meant by “prove” refers to phenomena such as overfitting, which can increase the risk of misinterpreting AutoML results. As such, the experts expressed concern that citizen developers entering with preconceived notions will constrain rather than broaden understanding of the problem. To address these risks, interviewees stressed the need for clear guidance on using AutoML tools, especially on communicating their limitations, including their capabilities and the data they require.

However, skepticism extends beyond citizen developers' capabilities or adoption of AutoML. Some AI experts questioned AutoML's effectiveness as a true boundary object. P11, an AI expert at a research center that collaborates closely with industrial partners to develop application-specific AI systems, emphasized that selecting the best-performing model should not be treated as the end of the development process:

“*So there exist [AutoML tool]. They try out 10 [models/algorithms] of them and they use the best ones from this. And they are producing good results. They would say ‘That is good for me. Fine. Done.' I don't see it like that.”* (P11)

P11's account highlights a concern that AutoML may encourage users to accept technically promising results without sufficiently examining the underlying data, model assumptions, or fit with the intended application. To navigate these challenges, fostering dialogue and establishing best practices for integrating AutoML into the development process will be essential.

### 4.2 Data handling

The data handling phase is considered the most labor-intensive part of AI development, where most challenges arise. Here, AutoML prototypes were proposed to serve as boundary objects, standardizing data inputs and translating domain knowledge into ML-compatible formats. As shared artifacts, they allow citizen developers to present their data in a way suitable for ML applications, while AI experts gain an initial understanding of the data's structure, quality and limitations. Drawing on many years of consulting experience with clients across different industries, P5 emphasized that data work often requires extensive discussion because client organizations differ widely in their data practices and levels of data maturity:

“*You see the data exactly in line with the way they [citizen developers] put it in[to the AutoML tool]. And you really have to ask, ‘Does this fit?' These are both technical and procedural aspects.”* (P5)

This account highlights that entering data into an AutoML tool does not in itself establish whether the data are appropriate for the intended use case. However, pre-defined data preparation steps can make relevant technical and procedural requirements visible and provide a common basis for evaluating data suitability. P5 further illustrated the practical challenges encountered when preparing organizational data:

“*People don't know how to work with data. Like you said, they somehow bind it in Excel, pull out whatever, stitch it together somehow, and in the end, well, that's how it looks.”* (P5)

Thus, by making data preparation more explicit, AutoML was perceived as helping both domain and AI experts identify problems early and refine datasets collaboratively. A similar view was expressed by P3, who also worked in consulting and regularly encountered varying levels of data maturity across client organizations. P3 described AutoML as providing an initial indication of data consistency and likely model outputs, while emphasizing that the data still require further review:

“*[…] how consistent the data is, and what kind of output they can expect from certain data during modeling. Maybe it gives an initial impression. But you still have to go over it again. It's more about tendencies–what works well and what doesn't.”* (P3)

Participants noted that the value of the prototype, therefore, lies not only in its assessment of the data but also in the discourse it enables. The exchange between citizen developers and AI experts, in which they discuss how data preparation steps affect results, offers the most profound learning opportunities and helps bridge knowledge gaps. It helps citizen developers understand the relevance of specific data points to achieving desired outcomes, while assisting AI experts in analyzing how these data points and preparation steps, like feature engineering, affect ML models and results. P2 stressed that business stakeholders need to remain actively involved in this implementation step:

“*As I said, there must be an understanding of the data on the business side. Of course, the business has a better understanding of the data than we do when we first come in, but they also need to be involved in this implementation step. There needs to be ‘citizen data science,' where you don't just throw everything into the [AutoML] model, but instead, you actively engage with it. You check if the model is really using data that makes sense, and you collaborate with the business to challenge the results, asking why certain factors have such a big impact.”* (P2)

Experts frequently highlighted that the data handling phase cannot be fully automated, as it often requires time-intensive collaboration to address challenges in data collection, processing, and quality assurance. Thus, this form of joint AutoML-supported exploration can make the effects of preparation steps more visible to domain stakeholders, helping AI experts identify contextual dependencies, assess data needs, and distinguish meaningful patterns from data artifacts.

Participants perceived the iterative use of AutoML as supporting learning across data-preparation activities. Initial model outputs and data-quality checks could indicate whether further collection, annotation, cleaning, or transformation is needed. Repeated engagement with the prototype may further foster learning across project iterations. Citizen developers could develop a more realistic understanding of the requirements for usable AI data, while AI experts can refine their understanding of domain-specific data practices. In this sense, AutoML was not perceived as eliminating data work. Instead, it was perceived as structuring collaboration around data work and supporting a more transparent division of responsibilities between domain and AI experts. Elaborating on these potentials in data handling, one AI expert clearly articulated the benefits:

“*... you can see that you need more data, or that you need to improve/enhance the annotation, effectively how to generate such actionable insights.”* (P1)

Participants perceived such feedback as potentially strengthening the impact of data quality, thereby motivating citizen developers to take a more active role in the data preparation process. Stronger involvement from citizen developers was perceived to foster more context-specific communication, improving data handling.

However, participants stated that these benefits depend on AutoML being embedded in a broader collaborative process. Data collection, processing, quality assurance, and the interpretation of outputs cannot be fully automated. Self-service access alone may therefore be insufficient, particularly when users lack experience with data preparation or when organizations have limited time and resources for exploratory work. Shared training, continued dialogue, and support from AI experts are necessary to ensure that AutoML outputs are interpreted appropriately and translated into productive next steps.

Participants anticipated that several risks could arise when this collaborative framing is absent. Poor-quality data may produce weak initial results, which citizen developers may interpret as evidence that a use case is not viable. Additionally, overexerting citizen developers was noted as a risk of not suitably utilizing AutoML prototypes and may cause them to lose motivation. Domain users often expect quick, actionable, and presentable results. As one expert explained, discouraging results generated with AutoML, particularly when rigorous tasks like data cleaning are not properly addressed, can have a negative impact:

“*Another thing is if we're just trying some standard approaches, maybe we can get discouraged by the initial results. So, if we run through the software and we say, okay, the initial results are quite bad, we can say, okay, it doesn't make sense to go further with. But maybe with some data preparation steps or putting some more thought into how can we model that or how can we adjust that to the problem, maybe we can get much more performance from this model.”* (P13)

The expert also highlighted that citizen developers could become frustrated, even if AI experts are able to improve results. In this regard, AutoML needs to be carefully utilized to truly function as a boundary object, supporting collaboration and learning without requiring extensive prior expertise. Establishing a process that enables mutual learning and communication at a manageable level is essential to ensure productive collaboration without overwhelming citizen developers.

### 4.3 Model building

Communicating results is a key challenge in the model-building phase. AutoML tools automate testing of standard AI models, display training results, and produce prototypes that benchmark what is achievable with the available data. In this way, AutoML prototypes were perceived as potentially helping bridge the knowledge gaps between AI and citizen developers, fostering further development and discussion of the AI system. The prototype provides citizen developers with insights into selecting ML models and hyperparameters. It facilitates learning for both domain and AI experts, with citizen developers receiving performance results, while AI experts can identify suitable models and discuss their feasibility for the given use case.

The prototype was anticipated to function as a shared artifact that provides an initial basis for discussing model selection, hyperparameters, and expected performance without requiring citizen developers to understand all underlying development processes. This accessibility was regarded as a central advantage of AutoML, particularly for stakeholders without deep technical expertise. However, the availability of results alone does not ensure that they can be understood or used productively. For AutoML prototypes to function effectively as boundary objects, results must be communicated in ways citizen developers can understand and apply. As one expert explained:

“*You can't assume that visualizations understandable to a data scientist will make sense to a process expert [...]. If I show them a boxplot, they'll ask, “What's the point of that? What should I do with this?” This translation is a challenge.”* (P6)

Several interviewees underscored that standard ML metrics and visualizations rarely meet the needs of citizen developers. AutoML results need to be translated into domain-specific, actionable insights to evaluate whether they fit their process, meet expectations, and warrant further development. P12 explained typical questions that they have heard regarding this issue:

“*What does it mean when I have an AI system and I get quality metrics established in the AI research field? But what does that mean for my company process? Does it fit? Is it sufficient? Can I apply it, or not?”* (P12)

The AutoML prototype was therefore perceived as potentially promoting discourse not simply by presenting results, but by providing a common reference point through which domain and AI experts could clarify what those results mean for the intended application. Such discourse can promote mutual learning. Citizen developers can better understand AI capabilities, limitations, and model trade-offs, while AI experts can clarify domain requirements, relevant performance criteria, and possible sources of observed weaknesses. The prototype may therefore help both groups identify areas for refinement rather than treating model performance as a purely technical outcome.

Integrating AutoML as a boundary object was also seen as a potential accelerator for the development process when the use case and data aspects have been refined in earlier phases. Some interviewees saw great potential in AutoML to accelerate early development, enabling rapid communication of preliminary results to clients. As one interviewee said:

“*I think with AutoML you are able to easily achieve faster good results and you have more things with the stable approaches so you will be able to explore which is time efficient [...] in the development, I think it can be easily integrated and then used to show, to communicate results with the client side.”* (P9)

However, AI experts cautioned that the focus should not solely be on speeding up result delivery, but also on critically evaluating whether the insights from the AutoML prototype are sound. Since AutoML tools automatically build AI models and benchmarks, both domain and AI experts must review outcomes to ensure their validity. One expert explained that the advantages of automation need to be considered carefully:

“*Of course, AutoML handles these steps automatically with standard procedures. But there should also be a step where someone reviews it and asks, ‘Does this actually make sense?'”* (P2)

This review is particularly important because apparently strong performance metrics may conceal unsuitable assumptions, insufficiently representative data, or decision thresholds that are not appropriate for the organizational context. They therefore viewed AutoML outputs as provisional inputs for joint interpretation rather than evidence that a model is ready for implementation. Participants perceived early proof-of-concept outputs as potentially enabling AI experts to discuss AI solutions and supporting learning about AI capabilities. In addition, AutoML allows AI experts to have more time to investigate and optimize AI systems:

“*The next question will rather be ‘Okay, can we include other data?' I always try to think from the business perspective. Can we try to better understand the processes? Okay great, are there other aspects where it also does not work? So, to look at instances where the predictions are not so good. To look into such aspects and say ‘Okay, what happened here, is there anything?' So, I would really just continue from this point and then say good, this would have been the first three cycles, my prototype phase and I continue from that.”* (P2)

An identified risk concerned the limited flexibility and transparency of standardized AutoML processes. Automated model selection and benchmarking were noted to obscure relevant contextual information, parameters, or non-standard development steps. Participants saw this as a potential decrease of AutoML's usefulness as a boundary object when stakeholders cannot understand or question how a result was produced, or when a use case requires approaches beyond the tool's capabilities. Additionally, non-standardized procedures often excluded from AutoML tools play a key role in achieving the desired outcomes in AI development projects. As one expert noted:

“*But if our business process or our processes are deviating significantly from the possibilities of the commercial off-the-shelf software, we need to go tailor-made.”* (P13)

While automation typically sacrifices some flexibility, most agreed the trade-off is acceptable for many cases, as one AI expert explained:

“*Automation takes away flexibility, this is obvious, this is how any automation works [...]. For many, many situations it does not matter. Here, the main point is that it gets faster and works better. But there are certainly edge cases when you need to, despite or precisely because of that, develop customized and individual solutions.”* (P12)

In such situations, reliance on the AutoML prototype was anticipated to create an overly narrow understanding of what is technically or organizationally possible. Interviewees suggested that AutoML prototypes could function as productive boundary objects only when standardized prototypes are embedded in a process of explanation, dialogue, and critical review. Clear communication of model outputs in domain-relevant terms, opportunities to discuss uncertainties and weaknesses, and the possibility of moving beyond standard procedures when necessary are essential conditions. Under these conditions, AutoML can support faster exploration and more effective collaboration without reducing model building to opaque or overly simplistic automated results.

### 4.4 Overarching aspects

Some recurring themes spanned all phases of AI development, underscoring the need to address these overarching aspects to effectively integrate AutoML as a boundary object. Interviewees agreed that access to AutoML offers little value without proper guidance for citizen developers. Its effective use requires a basic understanding of AI system development, rather than simply operating the AutoML tool. Training and education were deemed essential to effectively utilize AutoML prototypes as boundary objects. As one interviewee remarked, the mere availability of AutoML tools does not guarantee improved communication or collaboration:

“*Democratizing access does not mean democratizing knowledge.”* (P7)

While AutoML enables quick results with minimal effort from citizen developers, deeper explorations demand guidance and self-directed learning. AutoML tools themselves could aid this process, but ensuring citizen developers understand their prototypes remains crucial. This includes understanding that early prototype results do not necessarily demonstrate that an AI system will work reliably in practice. The interviewees acknowledged AutoML as a boundary object to engage citizen developers in AI development, but stressed the need to recognize its limitations. Ensuring AI quality is inherently complex and often slows deployment because it requires aligning domain requirements with rigorous testing. Participants thus noted that training should support a realistic understanding of prototype results, including their dependence on available data, modeling choices, and evaluation procedures.

Experts cautioned that excessive confidence in AutoML outcomes can create unrealistic expectations and hinder collaboration if citizen developers fixate on prototype results. Instead of fostering productive discourse and learning, this dynamic may create communication barriers, as one party might refuse to deviate from the prototype or fail to understand the system's limitations. One interviewee articulated this challenge:

“*The question is, how much do I trust my clients to understand? What might confuse them? And how far do they believe they know enough to decide what should happen next? I'm not saying clients are ignorant–not at all–but they often form ideas that are simply not feasible.”* (P3)

Effective communication, continuous collaboration and clear guidance were seen as essential to prevent misunderstandings and to ensure the development of fair, accountable, and transparent AI systems.

Appropriate data management structures are a prerequisite for integrating AutoML as a boundary object in the AI development process. Data acquisition remains a major challenge, involving requirements communication, quality assurance, and privacy concerns. The availability of suitable datasets was identified as the biggest constraint, often limited by complex or poorly integrated systems. Further, one expert noted that the lack of accessible, well-organized data repositories hampers the utilization of AutoML:

“*It's not like we currently have a data repository where a data scientist or a citizen data scientist can simply access the data and it works. Many systems, such as SAP, are widely used but not very open, making it difficult to retrieve data.”* (P2)

Active engagement between domain and AI experts using the AutoML prototypes could foster mutual learning. The interviewees reasoned that this collaborative, bottom-up approach improves acceptance, facilitates smoother integration into workflows, and enables citizen developers to evaluate system suitability and resource needs. However, the success depends on effective communication, customers' willingness to collaborate, and supportive organizational structures.

A recurring theme was the need to define responsibility for exploring AI use cases, with interviewees stressing the need for diverse stakeholder involvement rather than relying on a single individual. Organizations need to clarify who is responsible for initiating use-case exploration, supporting citizen developers, reviewing prototype results, and deciding whether to further develop, adapt, or discontinue a prototype. The interviews also highlighted that time constraints, especially in SMEs, often limit their ability to engage in exploratory work:

“*It must definitely be enabled by the company culture. In smaller organizations–speaking from our side–we don't even have the manpower or resources for too much exploration. What we do has to be extremely focused.”* (P1)

Developing supportive structures and offering guidance from AI experts were seen as promising approaches to address these constraints. For example, co-working with citizen developers on AutoML tools was mentioned as a way to build knowledge while streamlining future development processes. Such structures require time for exploration and collaboration, access to relevant expertise, and governance arrangements that ensure prototype results are reviewed before they inform consequential organizational decisions.

## 5 Discussion

This study provides initial insights on how AutoML can be integrated into the AI development process as a boundary object. Drawing on Wenger (2000) perspectives on artifacts, discourse, and processes, we explore how AutoML prototypes could manifest and influence communication, mutual learning, and collaboration between domain and AI experts. We demonstrate how the thoughtful integration of AutoML prototypes could help overcome key challenges of AI development. Specifically, we make two key contributions: (1) We develop a detailed understanding of AutoML's potential impact on the AI development process, showing that while AutoML's enactment as a boundary object can lower existing barriers and facilitate development, improper deployment can introduce new risks. (2) We demonstrate the applicability of Boundary Object theory for analyzing bottom-up AI development processes using AutoML. We theorize LCNC AutoML prototypes as boundary-negotiating objects that are shared and materially stable enough to coordinate work, yet interpretively flexible enough for different participants to negotiate the scope of a feasible ML problem, appropriate data and target, and acceptable model performance across early development.

Regarding the first contribution, we extend the literature by exploring the handover of the AI prototype from citizen developers to AI developers. Literature so far acknowledges the existence of a new AI development process based on LCNC AutoML and the involvement of citizen developers, but sheds little detail on how the interaction between citizen developers and AI experts is affected (Viswanadhapalli, 2025). Giraldo and Laso (2025) argue that AutoML research often focuses on technical performance, conceptual frameworks, or isolated prototypes. It pays less attention to the practical gap between citizen developers who create AI prototypes with LC/NC AutoML tools and developers who build, train, and tune machine-learning models through traditional workflows (Truss and Schmitt, 2024). To the best of our knowledge, this is the first study to explore the intersection between citizen developers and AI experts in LCNC AI development. Our findings show that communication barriers, often rooted in knowledge gaps, are among the most influential barriers in AI development. AutoML has the potential to bridge these gaps, both as a technical tool and as a prototype that acts as a translator between stakeholders. However, poorly managed integration poses risks, underscoring the need for careful AutoML integration. By presenting AutoML's dual role and its potential as a boundary object, we provide a nuanced understanding of its impact across artifact, discourse, and process dimensions as shown in Figure 2.

**Figure 2:**
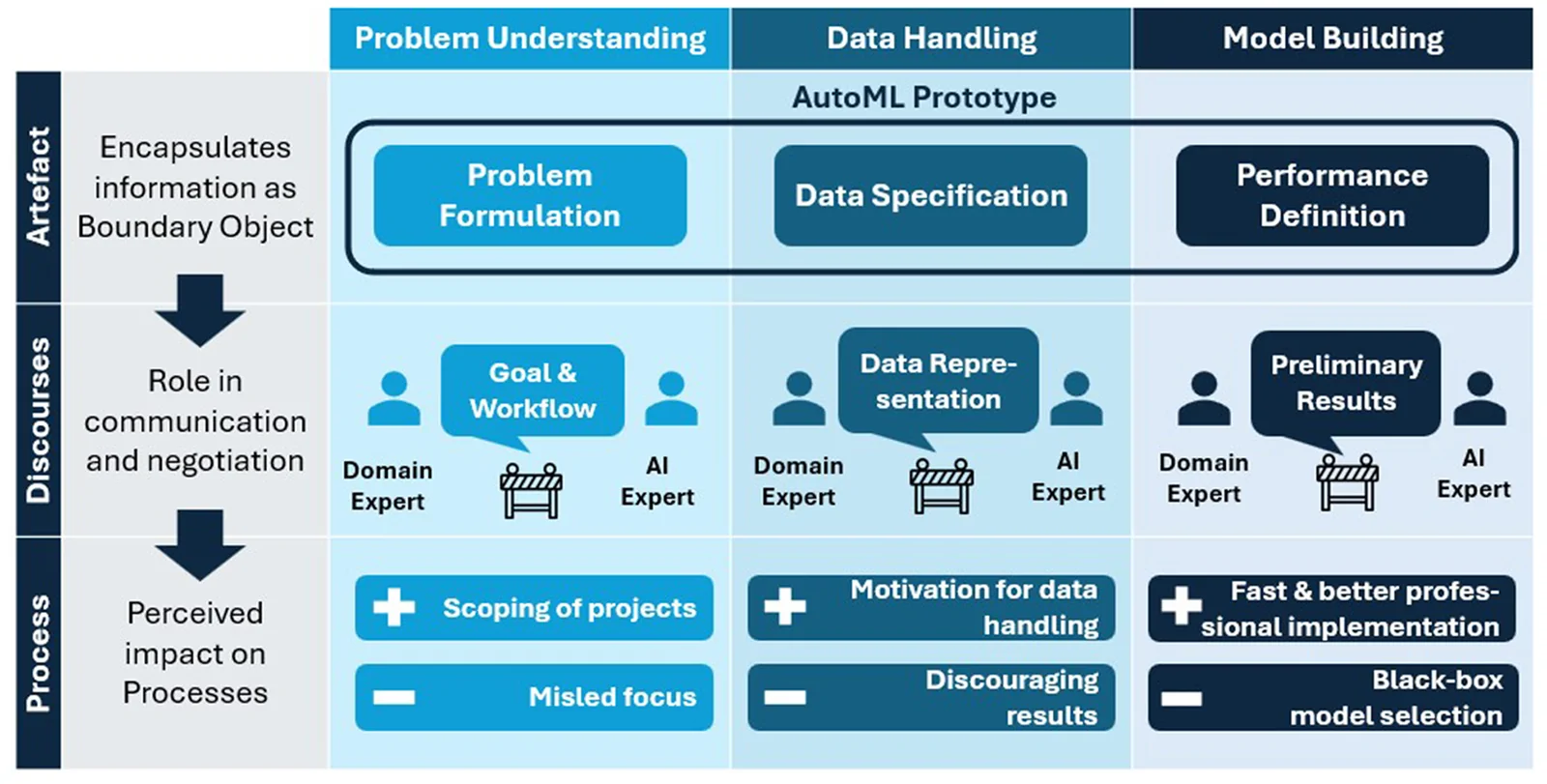
Summary of AutoML's perceived role as a boundary object.

From an *artifact* perspective, participants described AutoML prototypes as encapsulating the problem formulation, data specification, and the performance definition of an AI use case. Serving as boundary objects, they provide tangible frames of reference for basic AI capabilities, fostering discourse between the domain and the AI expert. Our findings indicate that AutoML prototypes aid feasibility testing, knowledge transfer, and structured communication. In the problem-understanding phase, AutoML prototypes establish a common language, helping to clarify targets and objectives. By doing so, they encapsulate the AI system's primary goal and structure the problem formulation. During the data handling phase, AutoML prototypes serve as boundary objects through pre-defined data formats, ensuring data quality that also helps encode domain knowledge. In the model building phase, AutoML prototypes play a crucial role in defining performance targets. They enable discussions on model suitability, performance transparency, and further refinements by facilitating model selection, performance benchmarking, and result visualization. Thus, AutoML prototypes structure key aspects of AI development, fostering collaboration while ensuring alignment between domain-specific requirements and AI capabilities.

From a *discourse* perspective, AutoML prototypes were seen to facilitate structured communication and foster mutual learning between domain and AI experts. In the problem-understanding phase, AutoML prototypes serve as tangible models that encapsulate the problem formulation within the tool's pre-defined workflow, establishing a common language and reducing communication barriers. They also mediate between stakeholder expectations of AI capabilities and limitations, enabling a more focused and precise problem discourse. During data handling, the AutoML prototype standardizes data representation by integrating domain-specific data into the AutoML tool. This structured approach fosters collaboration in exploring the role of data for model building. In the model-building phase, AutoML tools focus discourse on key performance aspects such as performance metrics and explainability approaches, while streamlining prototyping, model selection, and performance benchmarking. This fosters clearer communication, transparency, and alignment on emerging requirements.

From a *process* perspective, we found evidence that integrating AutoML prototypes as boundary objects enhances AI development by enabling feasibility checks, fostering collaboration, and creating early proof-of-concept prototypes. These benefits, however, depend on careful management. A structured problem-understanding phase, with clear encapsulation and discourse on problem definition, can help overcome communication barriers, while poor management can cause misaligned expectations. One of the most notable benefits is their potential to positively influence the data handling phase, supporting mutual learning, ensuring data relevance, and improving accuracy, provided that structured processes encourage active discourse. In the model-building phase, AutoML prototypes can streamline model selection, benchmarking, and development by accelerating collaboration between domain and AI experts. However, they also risk producing opaque “black-box” models that reduce transparency and hinder both the artifact's quality and the overall discourse.

### 5.1 Theoretical implications

Our second contribution is to conceptualize AutoML prototypes as potentially boundary-spanning artifacts that may support translation, negotiation, and learning between domain and AI experts. We examine how AI experts perceive AutoML prototypes as potentially functioning as boundary objects across the artifact, discourse, and process levels to support communication and collaboration among stakeholders. Boundary Object Theory suggests that artifacts become boundary objects when they are sufficiently plastic to accommodate different local interpretations while remaining robust enough to serve as recognizable points of reference across social worlds (Star and Griesemer, 1989; Akkerman and Bakker, 2011). In the context of AutoML prototypes, a shared dataset, target definition, model instance, evaluation output, and prediction interface can provide such common reference points. AutoML prototypes may therefore bridge knowledge gaps between domain and AI experts across the artifact, discourse, and process levels. Although the need for effective boundary objects in AI development is recognized, research on what constitutes and supports them remains limited (Truss and Schmitt, 2024). This study provides initial insights into how AI experts perceived AutoML's potential role in these dynamics.

Importantly, the value of AutoML prototypes does not lie in eliminating ambiguity. Rather, their value lies in making different perspectives visible and negotiable (Akkerman and Bakker, 2011). For example, a citizen developer may interpret an erroneous prediction as a business risk, or a violation of domain logic, whereas an AI expert may view the same case as a problem of data quality or model performance. The AutoML prototype can serve as a shared, interpretable object that helps stakeholders compare assumptions, clarify local knowledge, and negotiate decisions about feasibility, quality, and responsibility (Di Marco et al., 2011).

However, AutoML's boundary-spanning potential depends on the development context. In early, uncertain stages, prototypes may serve less as stable boundary objects and more as artifacts through which stakeholders negotiate shared meanings, roles, and practices (Lee, 2007). Their effectiveness thus depends on embedding them in supportive discourse and process structures that address practical challenges in collaboration (Al Alamin and Uddin, 2024). Consistent with prior work, we find that artifacts alone do not ensure collaboration; boundary spanners remain essential to mediate interactions (Levina and Vaast, 2005). Participants and prior literature suggest that any value attributed to AutoML prototypes depends on continuous discourse that exemplifies, contrasts, and frames ideas (Huber et al., 2020). This further reinforces the importance of citizen developers having basic AI knowledge to use AutoML effectively (Crisan and Fiore-Gartland, 2021), echoing Wang et al. (2019) view of AutoML as a learning tool.

More broadly, our findings support design and systems research showing that boundary objects alone cannot resolve issues of information sharing, team culture, and role clarity (Dolata and Crowston, 2024; Matthies et al., 2023). Experts in our study were open to integrating AutoML but demanded a clear definition of responsibilities and processes, echoing findings from studies that stress the need to facilitate discourse and manage processes effectively (Blomkvist et al., 2015; Holtmann et al., 2025). Building on research emphasizing the importance of upfront management for boundary objects (Wohlrab et al., 2019), AutoML similarly requires careful oversight. Overreliance on AutoML prototypes and blind trust in their results pose serious risks, underscoring the need for clear guidance. Overall, this highlights the need to rethink how AI development processes facilitate exchange between domain and AI experts, and to further investigate when, how, and by whom AutoML should be integrated to maximize its role as a boundary object.

### 5.2 Practical implications

First, our findings suggest that organizational LCNC development requires clear handover arrangements when citizen-developed prototypes are transferred to professional teams. As indicated in Truss and Schmitt (2024), after prototyping, a decision must be made whether to further develop the prototype with a professional AI team or to stop development. Our findings indicate that developing an AutoML artifact in isolation yields limited value, a well-documented issue in the broader artifact literature (Timperley et al., 2021). Thus, experts who interact with citizen developers are needed not only to guide go/no-go decisions but also to support the handover of promising prototypes to professional development teams. Thus, clear role definitions and supportive processes that align AutoML usage with organizational needs are essential for an organizational LCNC AI strategy forming the basis for coordination mechanisms such as shared mental models, effective communication, and mutual trust (Strode et al., 2022).

Our research shows that AutoML tools allow citizen developers to explore potential use cases, offering a hands-on approach that enhances understanding of AI capabilities and limitations. This early experimentation supports clearer decision-making on AI feasibility and cost-benefit considerations, addressing common barriers to AI adoption (Westenberger et al., 2022; Papagiannidis et al., 2023). In addition, prior research suggests that employee engagement, AI literacy, and continuous skill development are critical for reducing resistance and enabling positive workplace outcomes during AI adoption (Soulami et al., 2024). Organizations should thus clearly define the role of AutoML in their AI strategy, whether it is intended for AI capacity building, proprietary AI development, or broader process support. As Waardenburg and Huysman (2022) highlight, the lines between AI development and use are increasingly blurred, underscoring the need for early collaboration and open discussion between AI and citizen developers to align technical capabilities with business goals.

One of AutoML's most noteworthy advantages is that automation accelerates prototyping and model benchmarking, but must be carefully managed to avoid opacity, unrealistic expectations and overreliance on automated results. Critical AutoML research suggests that these tools are most valuable when they support a partnership between users and automation rather than attempt to remove human involvement from ML development altogether (Xin et al., 2021; Crisan and Fiore-Gartland, 2021). Citizen developers and AI professionals must still assess whether the data and ML targets represent the organizational problem, whether benchmark results are likely to generalize, and whether selected models are interpretable, fair, and acceptable for the intended use case (Sun et al., 2023; Quaranta et al., 2025). Without this review, AutoML may encourage overreliance on high-performing but unsuitable models. Human oversight is therefore essential when interpreting results and deciding whether to refine, hand over, or discontinue a prototype. At the same time, in AI development practices such as requirements engineering, AutoML shows promise for integrating human knowledge by helping teams incorporate it and discuss system behavior (Habiba et al., 2024). However, further research is needed to explore and validate these potential benefits.

To realize the benefits of AutoML effectively, organizations should establish specific roles, clear governance structures and training practices that define when and how AutoML should be used, prevent shadow IT, and align its application with strategic goals (Silic and Back, 2014). Such guidelines should include best practices, appropriate use cases, and mechanisms to align AutoML initiatives with organizational objectives. In addition, structured training can help citizen developers and AI professionals better understand the opportunities and limitations of AutoML, thereby reducing risks of overreliance or misinterpretation of results. Research on responsible AI adoption further emphasizes the importance of governance frameworks that support transparency, accountability, stakeholder collaboration, and ethical oversight throughout the AI lifecycle (Madanchian and Taherdoost, 2025). By embedding AutoML into structured, collaborative processes, organizations can improve project clarity, data quality, and development speed. With proper governance and education, AutoML can democratize AI adoption and drive digital transformation (Sundberg and Holmström, 2023).

## 6 Limitations and future work

First, this study focused on AI experts. While we know that citizen developers can create working prototypes with AutoML, we did not examine how they do so in practice. Because the study centered on the role of AutoML as a boundary object, future research should investigate citizen developers' practices when using AutoML.

One major limitation of this study is the limited geographic scope and composition of the interview sample. The sample was predominantly male and included a substantial proportion of participants in consulting roles. Although these participants contributed experience across multiple client organizations, their perspectives may place greater emphasis on project scoping, efficiency, and early prototyping. Future research should aim to include a broader, more geographically diverse sample to enhance the generalizability of the results. Additionally, we acknowledge that while translated quotes strive to convey the essence of original statements in German, they may not always capture their nuances perfectly. To address this, the authors employed a four-eyes principle, meticulously reviewing the translations to ensure accuracy and preserve the intended meaning.

Furthermore, this study primarily focused on the perspectives of AI experts. However, to better understand and explore how AutoML serves as a boundary object in practice, it would be valuable to conduct in-depth case studies that incorporate insights from citizen developers and end-users of the developed AI systems.

## 7 Conclusion

This study explored AutoML's potential as a boundary object in LCNC AutoML-driven AI development, focusing on its role in overcoming communication and collaboration barriers between AI developers and citizen developers. While prior literature centers on the technical capabilities of AutoML tools, their role in fostering collaboration and communication among stakeholders in AI development has been largely overlooked. By examining AutoML from this perspective, our findings provide initial insights into how AutoML might facilitate knowledge exchange and mutual understanding when effectively managed.

We contribute to a deeper understanding of AutoML's role in AI development by demonstrating that its enactment as a boundary object can reduce barriers and foster collaboration when managed appropriately, underscoring the value of Boundary Object theory for bottom-up AI development. Beyond analyzing AutoML prototypes as artifacts, our findings emphasize that democratizing AI development requires more than just providing citizen developers with accessible tools. It demands structured discourse and well-managed processes for meaningful engagement. AutoML's ability to generate actionable insights and early prototypes helps overcome data and modeling barriers while fostering iterative, feedback-driven development and improved outcomes.

Despite these promising implications, further research is needed to fully harness AutoML's potential in AI development. Moreover, understanding the broader societal implications of AutoML, including ethical considerations, biases, and accessibility, will be essential for shaping a responsible and inclusive AI landscape. By addressing these aspects, future research can unlock the full potential of AutoML while ensuring its responsible deployment in AI development.

## Data Availability

The datasets presented in this article are not readily available because consent for the analysis of the interview data was obtained only for this specific study and does not include the sharing of anonymised data. Requests to access the datasets should be directed to anna.polzer@uni-graz.at.
